# Dynamic hip screw in proximal femoral fractures followed by “single-stage” hip arthroplasty—retrospective analysis

**DOI:** 10.1007/s00590-023-03695-8

**Published:** 2023-11-08

**Authors:** Maros Hrubina, Libor Necas, Diaa Sammoudi, Juraj Cabala, Peter Lisy, Jozef Holjencik, Marian Melisik, Zoltan Cibula

**Affiliations:** 1 Jessenius Faculty of Medicine in Martin, Comenius University in Bratislava, Martin, Slovak Republic; 2 University Department of Orthopaedic Surgery, University Hospital Martin, Kollarova 2, 036 59 Martin, Slovak Republic

**Keywords:** Dynamic Hip Screw, Total hip arthroplasty, Proximal femoral fracture, Osteosynthesis failure

## Abstract

The aim of this study is to present the results of single-stage total hip arthroplasty (THA) after Dynamic Hip Screw (DHS) failure, or secondary posttraumatic osteoarthrosis. From 2003 to 2020, 15 THAs were performed in group of 15 patients for the treatment of DHS failure, or for late complications following femoral neck and pertrochanteric fractures. The mean follow-up period after arthroplasty was 46.9 months (range 7–139). The patients were evaluated retrospectively—both clinically and radiographically, focussing on the demography, infection rate and other complications (revision surgery), during the year 2023. 9 males and 6 females were included in the study, with a mean age of 56.5 years (range 29–93) at the time of primary osteosynthesis. Each of them had proximal femoral fracture treated primarily with a DHS and then late one-stage revision surgery, with hardware removal and THA implantation. The median time between DHS osteosynthesis and THA was 41.2 months (range 4–114). Four patients (26.6%) had complications after THA, with the need for revision in two cases (13.3%). Dislocation rate was 6.6% (one case), with the need for repeated-revision of THA. The infection rate was 6.6% (one patient) with the need for revision of THA. Peroperative periprosthetic femoral fracture was observed in 13.3% (two patients) without any other problems. Six patients (40%) died during the follow-up period. Single-stage total hip arthroplasty with concomitant hardware removal bears a high- mortality rate, with a higher incidence of postoperative complications compared to elective THA.

## Introduction

The incidence of proximal femoral fractures continues to increase, due to an ageing population, requiring osteosynthesis with devices, such as the Dynamic Hip Screw (DHS) [1]. With the increased number of DHSs implanted, the number of complications (failures, or secondary posttraumatic coxarthrosis) rises with the need for hardware removal and then revision by total hip arthroplasty (THA). Several complications (e.g. infection, hip dislocation) following these procedures have been described. The incidence of prosthetic joint infection (PJI) occurs in 0.2–2.2% after primary THA and about 9% in revision THA [2, 3]. This raises the question, whether to remove the hardware and then perform THA as a second stage, or to perform both a single-stage procedure. Some authors recommend a single-stage procedure, and the others warn against it due to increased incidence of PJI [4, 5]. Our University Department of Orthopaedic Surgery specialises in both primary and revision total hip arthroplasty. By the time we have performed a single-stage procedures after the DHS osteosynthesis in a relatively small group of patients. The DHS was used in the treatment of femoral neck fractures, or stable pertrochanteric fractures. Now, according to data from the Slovak Joint Arthroplasty Register, we have reviewed a group of patients after this type of proximal femoral osteosynthesis, who were treated for fixation failure, or secondary posttraumatic arthrosis, using single-stage THA.

The aim of this study was to evaluate retrospectively this cohort of our patients who had undergone THA (single-stage, with hardware removal) after DHS osteosynthesis, focussing on the complications and revisions.

Our hypothesis was that single-stage THA after DHS failure could lead to satisfied results comparable with other studies.

## Materials and methods

In this retrospective cohort study, the patients records were reviewed for demographic data, details of surgical procedures and follow-up results.

The study group consisted of 15 patients, whose proximal femoral fractures had originally been treated with DHS osteosynthesis, and who were than revised single-stage conversion to THA between 2003 and 2020.

This study was approved by our institutional review board.

### Inclusion criteria

Included in this study were 15 hips of nine males and six females, with a mean age at the time of the DHS osteosynthesis of 56.5 years. The primary diagnosis at the time of the surgery was of displaced femoral neck fracture (Garden type I–IV) or stable pertrochanteric fracture. Later, all these patients had single-stage THA with concomitant hardware removal.

### Exclusion criteria

Oncologic diseases, infection in the area of the operated hip joint, and patients treated with two-stage THA were excluded.

### Surgical procedures of THA

Preoperative planning and templating on radiographs were used to determine the implant sizes. In all cases, antibiotic prophylaxis was administrated prior to start the surgery (vancomycin 1 g parenterally and then every 12 h until the 5th postoperative day). All operations were performed via an anterolateral approach by surgeons specialised in revision arthroplasty.

The DHS was removed from the distal part of the wound, then total hip prosthesis was inserted.

In the cases with a cemented stem, we blocked the holes left after screw removal, using bone from the resected head, to prevent bone cement leakage into the lateral soft tissues. In THA with cementless stems, we used fluoroscopy preoperatively to prevent stem malposition, or undersizing.

The type of implants used were: cemented THA in three cases (Beznoska cup and stem—Kladno, Czech republic; Triloc cup, cemented Corail stem—DePuy, Warsaw, IN, USA), hybrid THA in one case (Pinnacle cup and cemented Corail stem—DePuy, Warsaw, IN, USA) and cementless THA in 11 cases (Delta PF cup, FIT stem, Minima stem—LimaCorporate, Udine, Italy; Pinnacle cup, cementless Corail stem, S-ROM stem, Tri-Lock stem—DePuy, Warsaw, IN, USA, Fixa Ti- por cup, Pulchra stem—Adler Ortho Spa, Milano, Italy).

### Postoperative management

Drainage was removed, and both active and passive movements started on the first postoperative day. Patients were discharged around eight days postoperatively (range: 6–15). Walking, touch-weight-bearing with crutches, was recommended until six weeks postoperatively, when patients were examined clinically and radiologically; thereafter partial weight-bearing was permitted. After the next clinical and radiological examination at the third month, full weight-bearing was allowed.

The patients were examined clinically and radiologically at six weeks, three months, six months, one year and then annually thereafter until the year 2023.

### Clinical outcome assessment

Basic demographic data were collected, including age, body mass index (BMI), affected side, type of proximal femoral fracture treated with DHS, reason of conversion to THA.

The clinical status of the patients was documented using the Harris hip score (HHS) [6].

We recorded early and late complications and any need for further THA revision.

Leg length discrepancy (LLD) was measured clinically and radiologically.

### Radiological assessment

On the preoperative radiographs (before the DHS osteosynthesis), we assessed the type of the fracture. On the radiographs before THA, we assessed the reason for THA implantation (posttraumatic arthrosis, femoral head avascular necrosis, implant cut-out phenomenon, and non-union). Radiological examination during the follow-up of the THA focussed on the loosening, defined as a periprosthetic radiolucent zone greater than 2 mm, or a migration greater than 2 mm with an adjacent radiolucent zone [7]. Heterotopic ossification was evaluated using the Brooker's classification [8].

### Statistical analysis

Two-sided, paired Student´s *t*-test was used for statistical analysis (after THA) of the pre- and postoperative Harris hip scores. Statistical differences were considered to be significant when the *p* value was < 0.05. A power analysis, using the R 3.5.0 (R Core Team, 2018), power package, indicated that a total sample of 15 patients (hips) would be needed to detect large effects (*d* = 0.8) with 80% power, using a two-sided paired *t*-test between means with alpha at 0.05 [9].

## Results

### Clinical analysis

Primary diagnosis of the surgery (osteosynthesis) was displaced Garden type I–IV femoral neck fracture in 10 cases (66.6%) and stable pertrochanteric fractures in 5 patients (33.3%). The mean age at the time of primary osteosynthesis was 56.5 years (range 29–93).

The left hip was affected six times, and the right hip nine times.

The mean BMI was 24.01 (range 19.60–30.97).

**Fig. 1:**
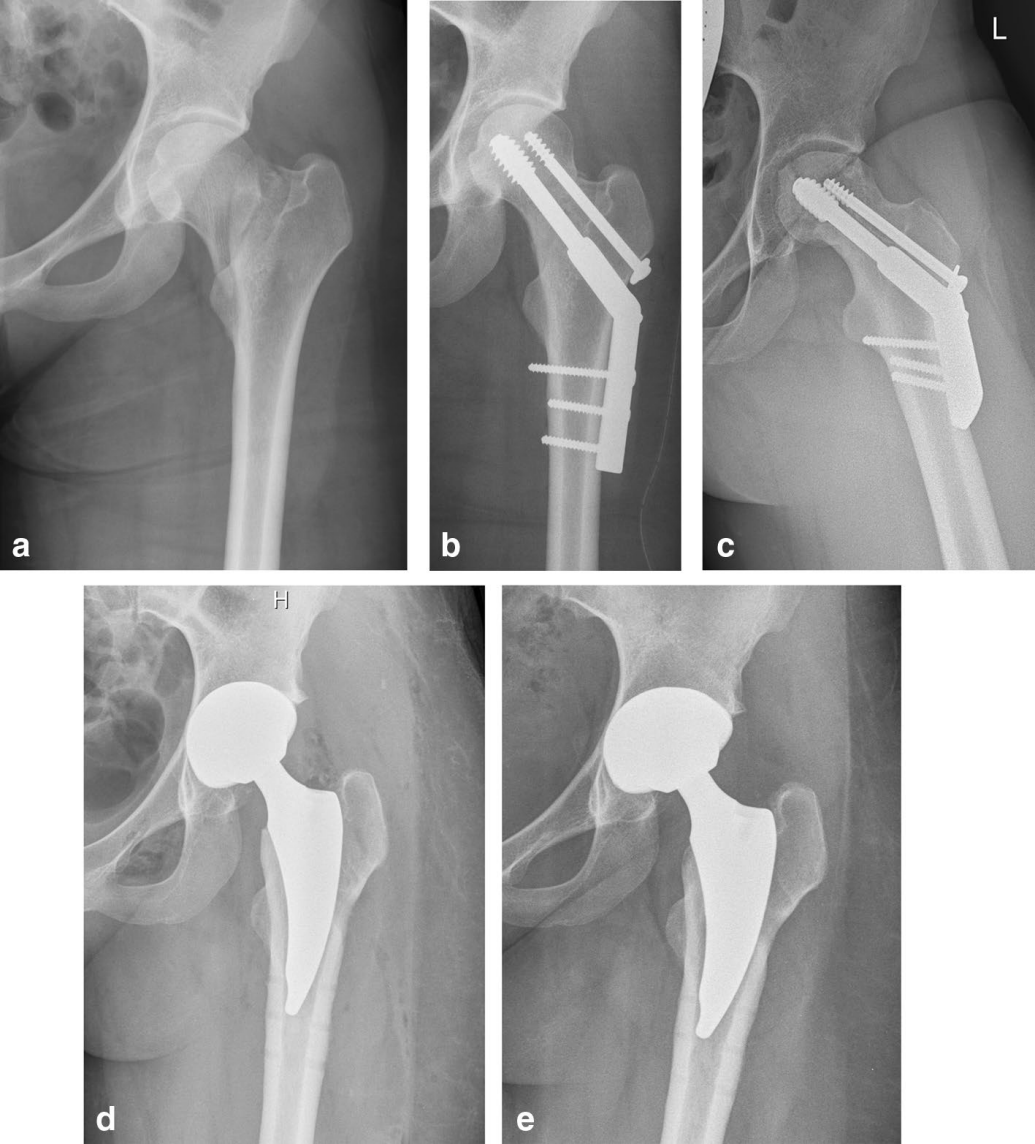
**a** Anteroposterior radiograph of 29-years-old patient with a displaced intracapsular femoral neck fracture Garden type IV of the left femur after the accident high energy trauma—fall from the horse. This case was fixed with DHS osteosynthesis within 6 h after the injury. **b** Anteroposterior radiograph of the same patient on the first day after the DHS osteosynthesis (with additional derotational screw), with correct fracture reduction and implant placement. **c** Axial radiograph of the same patient, 3 years postoperatively. The fracture is healed, but avascular femoral head necrosis (in the proximal part of the femoral head with visible collapse has occurred. Due to symptoms—increased pain and limitation of motion, the patient was allocated to single-stage THA following hardware removal. **d** Anteroposterior radiograph of the same patient on the first postoperative day after the DHS removal and THA (cementless THA with the short stem Minima, LimaCorporate, Udine, Italy). **e** Radiograph of the same patient three years after the THA. The Minima stem is in a neutral position with good osteointegration. The holes after the DHS are still visible

The reason for conversion of DHS to THA was avascular femoral head necrosis in nine cases (Fig. 1a-e), secondary posttraumatic coxarthrosis in three cases, cut-out phenomenon in two patients and femoral neck non-union in one.

The mean time from the DHS implantation to the THA was of 41.2 months (range 4–114).

The mean HHSs significantly increased from 14.2 (range 6–32 points) points before THA implantation to 78.4 (range 48–98 points) at the final follow-up (*p* = 0.001).

Finally, three patients (20.0%) had excellent hip scores (range 90–100 points), eight patients (53.3%) had good scores (range 80–89 points), three patients (20.0%) had fair scores (range 70–79 points) and one patient (6.6%) had a poor score (less than 70 points).

Average LLD was 3.6 cm shortening of the affected extremity before THA implantation (range 1.8–4.6 cm) and 0.5 cm average elongation of the operated extremity during the final follow-up (range 0–2 cm).

The mean follow-up period (after THA) was 46.9 months (range 7–139).

Patients´ data are listed in Table 1.

**Table 1:** Patients demographic and specification for single-stage THA implantation after DHS osteosynthesis

Patient	Age during DHS osteosynthesis (years)	Body mass index (BMI)	Affected side: right (R), left (L)	Type of fracture: femoral neck fracture (FNF), stable pertrochanteric fracture (SPF)	Time from DHS to THA implantation (months)/reason of revision	Type of used THA (fixation)	Complications	Revision surgery of THA (specification)
1	55	21.40	R	FNF	12/femoral head necrosis	Cementless	No	No
2	68	24.56	R	FNF	13/femoral head necrosis	Cemented	Perioperative periprosthetic femoral fracture	No
3	65	26.90	R	SPF	60/posttraumatic arthrosis	Cementless	No	No
4	45	23.45	L	FNF	8/non-union	Cementless	No	No
5	51	22.80	R	SPF	84/posttraumatic arthrosis	Hybrid	No	No
6	56	30.97	R	FNF	108/posttraumatic arthrosis	Cementless	No	No
7	58	28.00	L	SPF	114/posttraumatic arthrosis	Cementless	No	No
8	82	20.00	L	SPF	5/cut-out phenomenon	Cemented	No	No
9	62	24.50	R	FNF	38/femoral head necrosis	Cementless	No	No
10	31	25.25	L	FNF	54/femoral head necrosis	Cementless/short stem	No	No
11	66	23.20	R	FNF	30/femoral head necrosis	Cementless	Perioperative periprosthetic femoral fracture	No
12	93	22.00	R	SPF	4/cut-out phenomenon	Cemented	Repetitive luxations	Cup revision—reorientation with Dual—Mobility system
13	39	24.30	R	FNF	7/femoral head necrosis	Cementless	Early infection	Debridement, antibiotics, implant retention
14	29	19.60	L	FNF	36/femoral head necrosis	Cementless/short stem	No	No
15	48	19.60	L	FNF	45/femoral head necrosis	Cementless/short stem	No	No

Six patients (40%) died during the follow–up period (their mean survival time after THA was 26.8 months (range 7–52). Patient *n*°. 2 died for disseminated prostatic cancer 52 months after THA (aged 74). Patient *n*°. 3 died 7 months after THA (aged 71) from myocardial infarction. Patient *n*°. 6 died 42 months after THA (aged 69) from myocardial infarction. Patient *n*°. 7 died from unknown reasons 21 months after THA (aged 70). Patient *n*°. 8 died from terminal bronchopneumonia 16 months after THA (aged 84). Patient *n*°. 12 died 23 months after cemented THA, followed by revision for instability (aged 95).

### Radiographic analysis

The mean inclination angle of the cup was 44° (range 38–54°). We found neither migration of the cup, nor radiolucent lines around the cup.

We found no stem loosening. Stem alignment was neutral in all cases.

We found stem migration–subsidence to 2 mm with variation of 10° in one patient (*n*°. 14) with short cementless stem (6.6%) to 6th postoperative month. At the last follow-up (finally 60 months), this stem was osseously stable, without progressive variation, loosening or thigh pain.

Heterotopic ossification was diagnosed in five hips (33.3%)—Brooker type I was found in three patients, type II in one and type III in one.

### Complications and revisions

We found complications in a total of four patients after THA following DHS (26.6%), with the need for further revision in two cases (13.3%).

Two patients (*n*°. 2, 11) had perioperative periprosthetic femoral fracture treated with the use of cerclage wire, without any further problems during follow-up.

One THA *n*°. 12 dislocated repetitively, and was treated by closed reduction twice and then by further revision (2 months after conversion of DHS to THA), using cup reorientation with a dual-mobility system. This patient died almost two years after the last surgery.

One patient *n*°. 13 had early infection (S*staphylococcus aureus*) after THA, treated successfully by revision, debridement and implant retention on 16th postoperative day. During the last follow–up (82 months after the surgery) he was pain-free, without any signs of THA loosening.

## Discussion

Single-stage THA after DHS osteosynthesis failure, or for secondary posttraumatic coxarthrosis, is a relative uncommon procedure. Madariaga et al. describe only 15 cases from their whole cohort of 55 patients over 13 years. [1]. We have the same number of patients during 17 years. Some authors support that patients undergoing hip arthroplasty after failed osteosynthesis have a similar prognosis, namely with a notorious functional decline, increased number of hospitalisations with increased medical costs, as patients with proximal femoral fracture [10]. This type of THA is relative more difficult than primary THA, due to changes in the proximal femoral bone stock, the presence of the implant (DHS), which has to be removed, low bone mineral density, contracted soft tissues, and the deteriorating general status of the patient. This seems to be a predictive factor in the increased rate of complications following these procedures.

In our group of patients we had only one infection (6.6%) and a total of 26.6% complications. This correlates with the literature, where PJI after elective THA is lower [11]. The revision rate of THA in our group of patients was 13.3%.

The described re-revision rate for patients who had undergone a revision THA after failed osteosynthesis was three times higher than the rate of revision after a primary THA [12].

Some controversy is around whether hardware removal and the THA implantation in single- or two-stage procedure (hardware removal and THA implantation later as a second stage). Parvizi et al. [3] did not clarify which is the best approach regarding THA with concomitant hardware removal.

We have recorded 6.6% of early PJI and dislocation rate was of 6.6%. Madariaga et al. described an incidence of 9.1% for each of the above-mentioned complications [1].

In the use of cemented THA, there is a possibility to extrude the cement through screw holes [13]. Due to this risk factor and our increasing experience with the use of cementless THA (especially short conservative stems) we started to use non-cemented THA in these cases [14, 15].

The correct indication for the primary treatment of proximal femoral fracture is absolutely essential, with the focus on the minimalising complications [16]. But some complications will inevitably occur [17, 18].

DHS revision is a relative rare procedure. Akgul et al. described only 7 cases of failed DHS converted to a THA. The major reason for failure was avascular femoral head necrosis after femoral neck fracture osteosynthesis in 50% [19]. In our series it was 60%. On the other hand, Taheriazam et al. described 203 cases of failed DHS for intertrochanteric fractures treated by single-stage THA with a relatively low rate of complication (infection in 0.98%, dislocation in 0.49%) [20]. We now use proximal femoral nail (PFN) in intertrochanteric femoral fractures treatment primarily. On the other hand, DHS is now relatively rarely used due to the increasing number of PFN used for similar fractures, the complications are now less presented. However, in large published series of patients, the DHS results are similar to the results following PFN [21].

Our study has some limitations. First, the study was retrospective in design, not randomised, with a limited number of patients and using no control group. Studies performed over a long period of time (in our study 17 years) may suffer from variations regarding daily practice and protocols. Our group of patients is relatively small and heterogeneous due to: primary diagnosis of proximal femoral fracture, reason of DHS conversion and the implants used.

A second limitation is that different surgeons participated in the study, and this may present diversity in their intraoperative management and final results.

## Conclusion

DHS osteosynthesis failure is now a relatively rare occurrence, but it does still occur. Our study shows, that single-stage THA with concomitant hardware removal could lead to satisfactory results, which could be compared with the other published studies. The complication rate was 26.6%, with the need for further revision of the THA in 2 patients (13.3%).

Larger, prospective, randomised, multi-centre studies comparing both single versus two-staged THA after DHS failure need to be undertaken to determine the optimal strategy of treatment.

## Data Availability

Data and material available on request.
